# Trends and factors associated with long-acting reversible contraception in Kenya

**DOI:** 10.12688/f1000research.23857.1

**Published:** 2020-05-20

**Authors:** Wambui Kungu, Anne Khasakhala, Alfred Agwanda

**Affiliations:** 1 Population Studies and Research Institute, University of Nairobi, Nairobi, Kenya

**Keywords:** LARC, Benefits, Highly Effective, Predictors, Unintended pregnancy

## Abstract

**Background**: Kenya has 12 million female adolescents and youths aged 10-34 years whose reproductive behavior will determine the growth and size of its population for the next decade. The anticipated momentum of births can be slowed by the use of long-acting reversible contraception (LARC) methods as they are more effective, need no user adherence, and hence have no risk of incorrect or inconsistent use. However, in spite of the many health and social benefits, LARC is underutilized because of myths and misconceptions. Kenya is in the ultimate decade towards Vision 2030 and investing in LARC can save costs of health care and accelerate the achievement of the development goal. The objective of this study was to establish factors associated with LARC use, with a view of establishing the potential for increasing demand.

**Methods**: The study was national and used secondary data from the three waves of the Kenya Demographic Health Survey from 2003, 2008/09 and 2014 in a sample of all women of reproductive age who reported currently using modern contraceptive methods at the time of interview. Descriptive and logistic regression analysis was employed to profile and examine LARC users.

**Results**: LARC use was low but picking up rapidly, especially among contraceptive users of higher social economic status in a major shift between 2008/09 and 2014. Consistent factors that influenced its use were age, wealth, and number of living children, while education and residence were of influence some of the time.

**Conclusions**: There is huge unexploited potential for more LARC uptake based on the identified predictors of its use. Scaling up of LARC uptake is critical to deal with issues of poor user adherence, incorrect and inconsistent use, and method failure that characterize short-acting contraception, resulting in increased unintended pregnancies, incidences of unsafe abortions and maternal and infant mortality.

## Introduction

Family planning is a critical component of Kenya’s development agenda and it is addressed in the Vision 2030 social pillar on provision of reproductive health for the poor and vulnerable population. Kenya is a model of fertility transition, having moved from a very high level of fertility of over eight children in the 1970s to about four children in 2014, with a contraceptive prevalence (CPR) of 58%. A major recent growth in CPR was especially evident in the use of modern methods between 2003 and 2014. CPR increased by 17% between 2003 and 2014, while use of modern methods, which has driven the CPR up, increased by 21% over the decade ^1, 2^. The increased demand for contraception was generated by robust campaigns that were initiated with the repositioning of the family planning program. The agenda of family planning is also central in the International Conference on Population and Development after 25 years (ICPD25) Commitments as well as in Sustainable Development Goal (SDG) 3.7 ^3^.

Investing in family planning is highly cost-effective and can lower healthcare costs and cascade benefits to Kenya in an unrivalled manner. As of 2015, Kenya had saved US$4.48 directly in healthcare costs for every US$1 that was spent on family planning. If county governments accelerated demand and hence uptake of modern contraceptive methods, the savings would rise to US$5.46 for every US$1 used in family planning and result in a direct total saving of US$80 million by 2020 ^4^.

Long acting reversible contraception (LARC) refers to contraceptive methods that can be used beyond one year upon insertion and comprises the implant and intrauterine devices (IUDs). Evidence from the 2014 Kenya Demographic and Health Survey (KDHS) shows increased use of LARC in comparison to other modern contraceptive methods, with the implant becoming the second most popular method after injection ^1^. The reproductive behavior of the 12 million female adolescents and youths aged 10–34 years ^5^ will determine the growth and size of the population of Kenya for the next decade.

The anticipated momentum of births from the huge female population can be slowed by use of LARC as these methods are longer acting, reversible, more effective, need no user adherence and hence have no risk of incorrect or inconsistent use common to young contraceptive users and have lower rates of discontinuation ^6^. They are also credited with higher user acceptability/satisfaction, fewer side effects and user contraindications as well as little partner involvement. They are estrogen free, hence pose fewer risks to a woman’s health, and involve less visits to health facilities, hence less strain on the health system ^7, 8^. Despite these great health and social benefits, LARC is underutilized because potential users are not comprehensively counselled about them and additionally, the methods are initially expensive. Evidence on cost has, however, shown they are cost effective in the long-term and can result in savings of $12,000 in five years ^9^. In concurrence, Trussel *et al.* ^10^ found cost savings can be realized from LARC use within three years whether or not the methods are used to the end of their efficacy period. They arrived at costs of $304 for the IUD and $308 for the implant per woman, per annum in the United States.

Evidence has shown that unintended pregnancies and induced abortions could be considerably reduced by as much as 25% if women used more effective methods and specifically LARC ^11^. Using KDHS 2003 data, the study found a 0.4% failure rate for long acting methods against 2.7% for short acting methods and 15.8% for traditional methods. Total unintended births were 44%, out of which 5% were terminated. The number of unintended births that could be averted was estimated at 11% (69,000) out of the 44%. The expansion of access to LARC is critical in family planning programs that aim to address high rates of unintended pregnancy and curb high unmet need for family planning ^12^.

The current Kenya Family Planning Costed Implementation Plan (FP CIP) has several targets towards rights-based family planning and improved maternal and child health outcomes for the period 2016 to 2020. The targets are; increasing implant use from 12% to 16%; increasing IUD use from 5% to 7%; avert 2 million unintended pregnancies, 62,500 unsafe abortions, 4700 maternal deaths and reduce unmet need from 15% to 13%. An underlying target is to increase modern contraceptive prevalence (mCPR) from 54% to 58%, accompanied by high quality information and contraceptive services ^13^.

A challenge in the access to contraceptive commodities is that the method mix in Kenya is dominated by the injection, and increasing the share of LARC can reduce this domination as well as expand the method mix. Method mix heavily influences rates of method failure because short acting methods have higher failure rates as they are less effective. Evidence has shown that women are more likely to choose more effective methods if exposed to information and services about them ^14, 15^.

In contraceptive use dynamics, the role of method choice is critical in gauging the quality of services because client satisfaction, acceptance of a method and its continuation depend on it ^14^. The convenience, effectiveness and availability of contraception methods vary, hence the side effects likely to be experienced by different users and resultant discontinuation rates vary by method and also by user characteristics. It is therefore crucial for managers of family planning programs to ensure clients use the most suitable method to enhance continuity.

LARC use has only picked up in the last decade and there is still a paucity of data and a lot of barriers to its uptake. Evidence on predictors of its use is critical in the next decade of promotion of LARC methods to overcome the challenges that hinder women from enjoying the many benefits of LARC.

## Objective

The objective of the study was to establish the factors associated with LARC methods with a view to establishing potential for increasing demand for LARC.

## Methods

### Ethical statement

Specific ethical approval is not required for re-analysis of DHS data, but permission to use the data for this study was obtained from ICF. This study uses existing KDHS data and re-analysis was done under the original consent provided by the participants.

### Data sources

The study was national and used secondary data from the three waves of KDHS 2003, 2008/09 and 2014, mainly from the contraceptive calendar contained in the woman’s questionnaire for all eligible women and also the household file and individual woman file. The sample was all women of reproductive age, 15–49 years, who reported current use of any of the major methods of modern contraception at the time of the interview, whether married or not. Women who did not report current use of the modern contraception were excluded. Full details of sampling procedure, data collection procedure and variables for which data were collected are available in the Kenya DHS Final Reports ^16– 18^.

### Data variables

The dependent variable of interest was the current method of contraception, which was categorized as LARC if the method was IUD or implant or ‘other modern’ if the method was condom, pill, injection or sterilization. The independent variables selected were woman-level characteristics of age, education, marital status, number of living children, desire for children and household level characteristics of place of residence, wealth status, type of contraceptive region. Age was grouped into three 10-year categories (15–24, 25–34, 35+) while education was also grouped into three categories (none, primary, secondary +). Marital status categories were two (married/not married) and those of number of living children were four (none, 1–2, 3–4, 5+). Desire for children was either that the woman wanted more children or did not want more children, while place of residence was either rural or urban. Wealth status reclassified the five DHS categories as follows; lower (lowest/low), middle (middle) and higher (high/highest) while type of contraceptive region was classified into two categories; high contraceptive (Nairobi, Central, Eastern) and low contraceptive (the five remaining regions).

### Data analysis

The first stage of analysis profiled the sampled women using cross-classification analysis by their background characteristics. An analysis to establish the differentials of LARC against other modern methods based on the independent variables was conducted where the chi-square test identified if there existed any statistical significance. The confidence level was set at 95%.

The dependent variable, contraceptive method, was binary in two categories of LARC or ‘other modern’, hence the binary logistic regression model was employed to determine any influence of the independent variables on modern contraceptive method choice. LARC use was the outcome of interest, therefore ‘other modern’ was used as the reference category in the regression. The independent variables were recoded into fewer categories for ease in the analysis.

The software used for the data analysis was SPSS version 22.

## Results

In an attempt to obtain the profile of the LARC user, cross tabulations were undertaken to show the share of LARC use against other modern contraceptives and examine any significant relationships between the socio demographic factors and use of modern methods categorized under LARC or ‘other modern’ for each year under study. Table 1 presents the results.

**Table 1.** Differentials of modern contraceptive use for women aged 15–49 by various background characteristics in 2003–2014.

	2003		2008/09		2014	
N	1853		2196		10,976	
Variable	%		%		%	
	LARC	Other modern	LARC	Other modern	LARC	Other modern
**Age**						
15–24	7	93	3	97	19	81
24–34	13	87	11	89	26	74
35+	16	84	13	87	23	77
**X ^2^=18.071 P=. 000**			**X ^2^=35.796 P =. 000**		**X ^2^=35.083 P =. 000**	
**Education**						
None	6	94	5	95	22	78
Primary	7	93	6	94	22	78
Secondary+	13	87	15	85	26	74
**X ^2^=67.080 P =. 000**			**X ^2^=57.057 P =. 000**		**X ^2^=19.854 P =. 000**	
**Residence**						
Urban	14	86	14	86	27	73
Rural	11	89	7	93	21	79
**X ^2^=3.767 P =. 052**			**X ^2^=34.577 P =. 000**		**X ^2^=37.970 P =. 000**	
**Wealth**						
Lower	6	94	4	96	20	80
Middle	8	92	7	93	22	78
Higher	16	84	13	87	27	23
**X ^2^=29.848 P =.000**			**X ^2^=37.222 P =. 000**		**X ^2^=54.427 P =. 000**	
**Region**						
High contraception	10	90	9	91	23	77
Low contraception	13	87	10	90	24	76
**X ^2^=4.039 P =. 044**			**X ^2^=.678 P =. 410**		**X ^2^=.697 P =. 404**	
**Marital status**						
Married/living together	13	87	11	89	24	76
Not married/living together	7	93	6	94	20	80
**X ^2^=9.327 P =. 002**			**X ^2^=7.051 P =. 008**		**X ^2^=21.078 P =. 000**	
**No of living children**						
None	4	96	3	97	3	97
1-2	14	86	11	89	25	75
3-4	13	87	12	88	25	75
5+	10	90	7	93	23	77
**X ^2^=11.016 P =. 012**			**X ^2^=17.186 P =. 001**		**X ^2^=115.527 P =. 000**	
**Desire for children**						
Wants	11	89	9	91	22	78
Does not want	13	87	10	90	24	76
**X ^2^=1.149P =.284**			**X ^2^=1.839 P =. 175**		**X ^2^=3.750 P=. 053**	

**Notes: P-value= 0.05.** **LARC, long-acting reversible contraception.**

Table 1 shows low shares of LARC, ranging from 5% to 15% among modern contraception in 2003, reducing slightly in 2008/9, before a 2-6 fold increase across all socio demographic factors to reach 20–26% in 2014. Other modern contraception had huge shares of 85–95% in 2003 and 2008/09, which declined to 75–80% in 2014. For women with no children, LARC use was very low at 3% across the data sets. The emerging general profile of the LARC user was that of secondary educated, mostly married women of higher wealth, living in urban areas, with at least one child and aged 25 to 34. All the factors apart from region and desire for children exhibited significant relationships with use of modern methods.

### Levels and trends in LARC use

**Figure 1.**
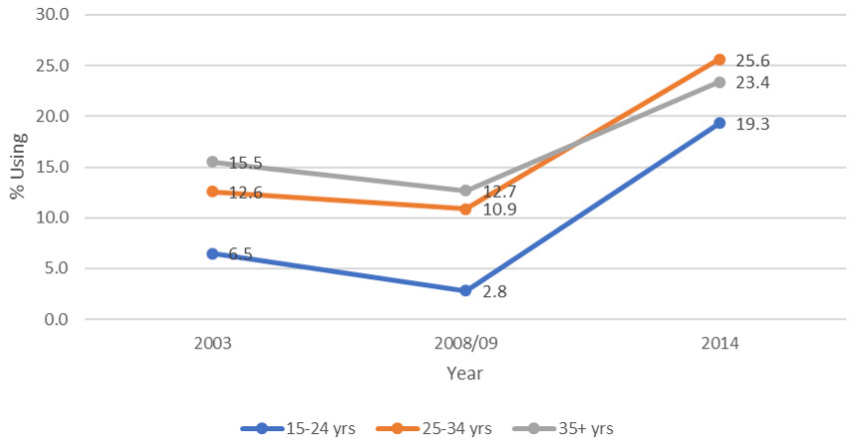
Long-acting reversible contraception use by age in Kenya, 2003–2014.

To find out levels and trends in LARC use against other modern methods, descriptive analysis was done based on age and regions. Figure 1 to Figure 9 present the different results. Figure 1 complements Table 1 results on age.

**Figure 2.**
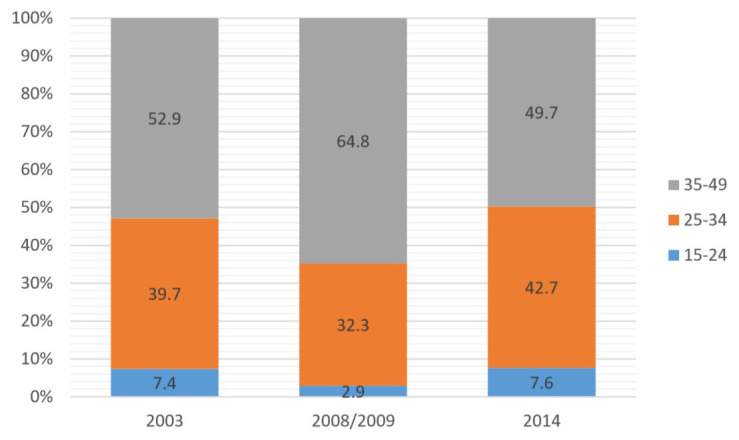
Intrauterine device use by age, 2003–2014.

**Figure 3.**
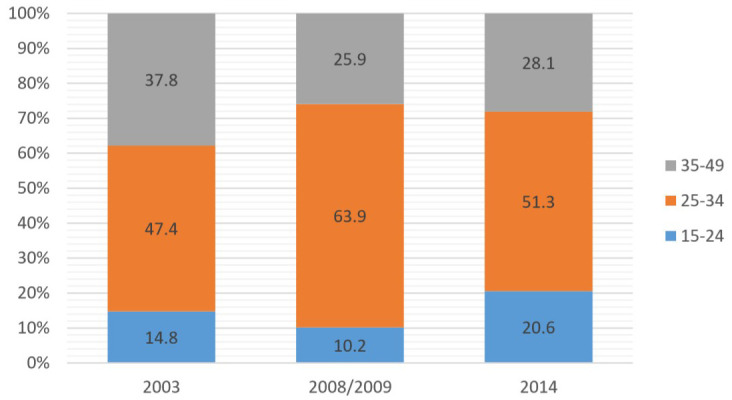
Implant use by age, 2003–2014.

**Figure 4.**
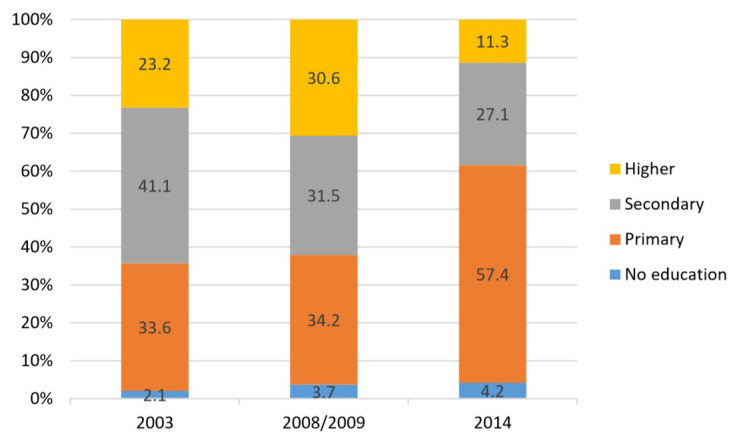
Implant use by education, 2003–2014.

**Figure 5.**
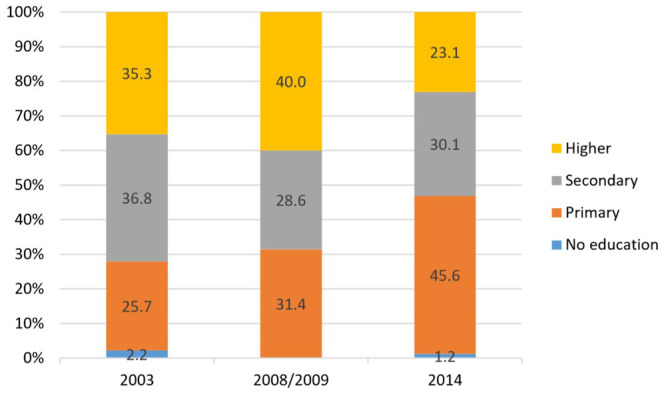
Intrauterine device use by education, 2003–2014.

**Figure 6.**
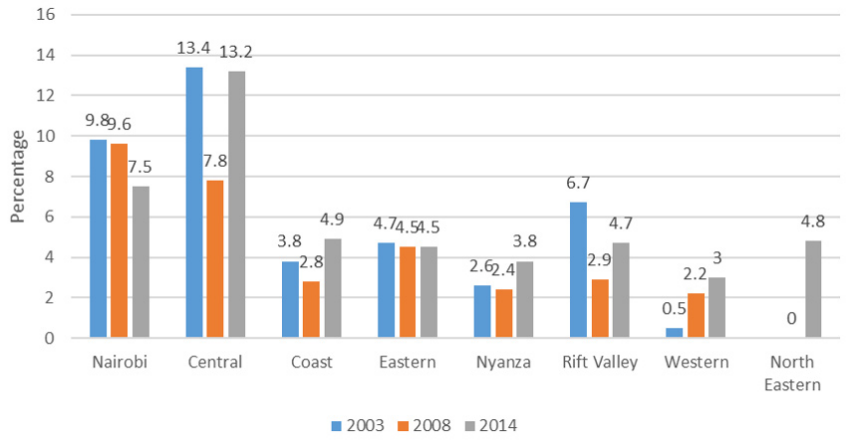
Percentage use of the intrauterine device among modern contraceptives by region, 2003–2014.

**Figure 7.**
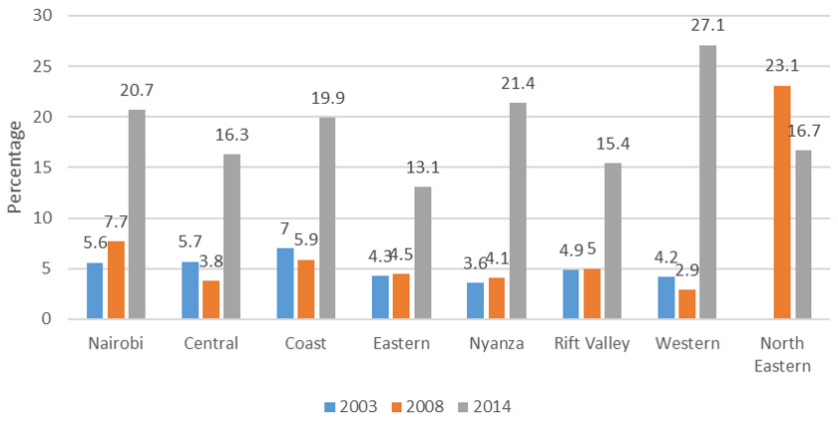
Percentage use of implants among modern contraceptives by region, 2003–2014.

**Figure 8.**
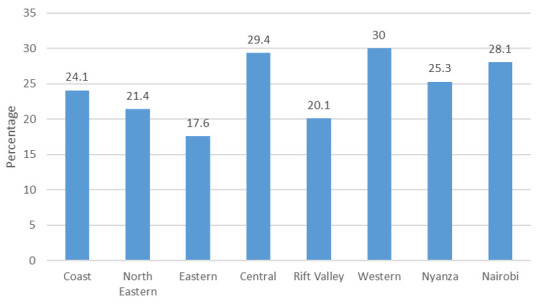
Percentage use of long-acting reversible contraception among modern contraceptives by region, 2014.

**Figure 9.**
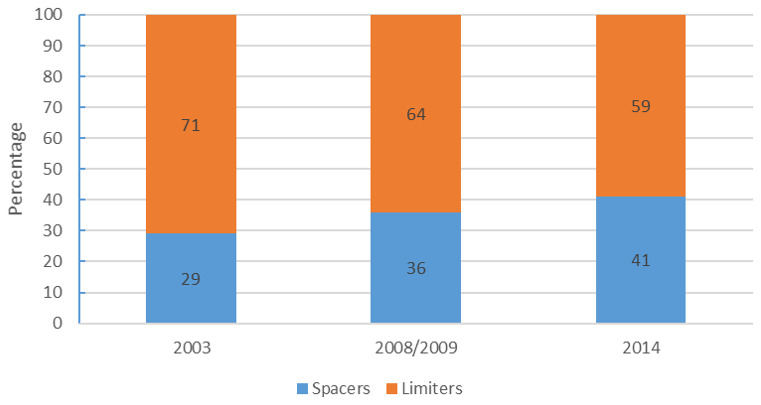
Trends in long-acting reversible contraception use by fertility desire, 2003–2014.

***LARC use by age***. Figure 1 shows LARC use in 2003 and 2008/09 was highest among women aged over 35 years, but in 2014 was highest among those aged 25–34. There was a decline in LARC use between 2003 and 2008/09, and then major surges between 2008/09 and 2014. Use among 25–34 and 35+ age groups each increased two-fold, while for the 15–24 group, the increase was seven-fold.

Further analysis (shown in Figure 2 and Figure 3) on age showed that for the IUD, the majority of users were in the 35–49 age group, while for the implant, the majority were in the 25–34 age group. The lowest use for both was among the 15–24 age group.

***LARC use by education***. Analysis by education (shown in Figure 4 and Figure 5) revealed that in 2014, the majority of users for both IUDs and implants, with a 50–60% share each, had primary level education, while the lowest use for both was among women with higher education.

***IUD use by region***. Figure 6 shows that the share of IUD use among modern contraceptive use in the Central region was highest in 2003 and 2014 at 13%, while its share in Nairobi was highest in 2008/09 at around 10%. The North Eastern region had 5% of its modern contraceptive users using the IUD in 2014. There was no data for the region in 2003 and there were no IUD users in 2008/09. The Eastern region showed consistent use, with 5% of its modern method users using the IUD, while the Western region showed a gradual rise in IUD use across the three data sets. Results between 2008/09 and 2014 show an increase in IUD use in all regions except for the North Eastern region, with the biggest change being in the Central region, where IUD share among modern methods increased two-fold from 3.2% to 6.3% in prevalence.

***Implants use by region***. Figure 7 shows the low share of implants among modern contraceptive users in 2003 and 2008/09, before a major rise in the share of implants across all regions in 2014. The Western region in 2014 led, with 27% of its modern method users using the implant, followed by Nyanza, Nairobi and Central, each with around 20% share.

***LARC use in 2014***. The status of LARC share among modern contraception in 2014 in all regions is shown in Figure 8.

Figure 8 shows the level of LARC use after the surge that occurred between 2008/09 and 2014, wherein the share of LARC among modern contraceptive use rose to comprise 20% to 30% of the share of modern contraceptive use in all regions apart from the Eastern region. The Western region, which earlier had very low shares of IUD and implant use, caught up with Nairobi and Central to reach a 30% share for LARC among modern contraceptive users.

***Trends in LARC use by fertility desire***. Another analysis done on LARC users was by fertility desire (whether one was spacing or limiting children) in an attempt to establish whether family planning clients were using the appropriate methods for their reproductive goals. The results are presented in Figure 9.

The results show that majority of LARC users are limiters in all the data sets but there is a sustained total decline of 12% from 71% in 2003 to 59% in 2014. Running counter to this is an equal increase among spacers from 29% in 2003 to 41% in 2014.

### Regression analysis

For the regression analysis, the dependent variable was modern contraceptive use with ‘other modern’ as the reference category. Reference categories for the independent variables are indicated for each variable. The results of the regression analysis are presented in Table 2.

**Table 2.** Logistic regression results for LARC use for all women of reproductive age in Kenya 2003–2014.

Variable	2003			2008/09			2014		
	B	SE	EXP B	B	SE	EXP B	B	SE	EXP B
N	1853			2196			10,976		
**Age**									
15–24 **(Ref)**	.000		1.000	.000		1.000	.000		1.000
24–34	.644	.255	1.905 ^*^	1.265	.306	3.542 ^***^	.140	.067	1.150 ^*^
35+	1.089	.298	2.970 ^***^	1.591	.337	4.909 ^***^	-.035	.082	.966
**Education**									
None **(Ref)**	.000		1.000	.000		1.000	.000		1.000
Primary	.123	.490	1.131	.064	.538	1.066	-.009	.126	.991
Sec+	1.016	.488	2.762 ^*^	.839	.541	2.313	.187	.131	1.205
**Residence**									
Urban **(Ref)**	.000		1.000	.000		1.000	.000		1.000
Rural	-.226	.168	.797	.347	.179	1.415	.200	.051	1.221 ^***^
**Wealth**									
Lower	-.784	.270	.457 ^**^	-.792	.293	.453 ^**^	-.298	.062	.743 ^***^
Middle	-.545	.242	.580 ^*^	-.292	.239	.747	-.202	.063	.817 ^**^
Higher **(Ref)**	.000		1.000	.000		1.000	.000		1.000
**Region**									
High **(Ref)** Contraception	.000		1.000	.000		1.000	.000		1.000
Low Contraception	.250	.188	1.284	.092	.177	1.096	.062	.049	1.064
**Marital status**									
Married/living together	.395	.242	1.484	.274	.236	1.316	.030	.065	1.031
Not married/living together **(Ref)**	.000		1.000	.000		1.000	.000		1.000
**No. of living children**									
None **(Ref)**	.000		1.000	.000		1.000	.000		1.000
1–2	1.268	.625	3.555 ^*^	1.098	.550	2.997 ^*^	2.397	.278	10.990 ^***^
3–4	.932	.652	2.540	1.196	.578	3.308 ^*^	2.495	.284	12.127 ^***^
5+	.697	.681	2.008	.792	.623	2.208	2.553	.291	12.846 ^***^
**Desire for children**									
Wants **(Ref)**	.000		1.000	.000		1.000	.000		1.000
Does not want	-.097	.190	.907	-.171	.191	.843	.063	.062	1.065

**Notes:** P-value = 0.05 <.05* < .01** <.001 ***. Ref, reference category; LARC, long-acting reversible contraception; B, Beta coefficient; SE, Standard error; EXP B, exponentiation of the B coefficient (odds ratio).

### Determinants of LARC use

Three consistent predictors of LARC use emerged across the study period. These were age, wealth and number of living children. Education was a predictor in the 2003 data set, while residence was a factor of influence in the 2014 data set.

Regarding age, in 2003, it showed little influence among the 25–34 age group, while for the 35+, its influence was very strong. Women aged 25–34 were almost twice as likely to choose LARC than those aged 15–24, while those aged 35 years and above were almost three times more likely to choose LARC than the 15–24-year-olds. In 2008/09, the influence was very strong in both age groups. Women aged 25–34 were 3.5 times more likely to use LARC than their younger counterparts, while those aged at least 35 years were almost five times more likely to choose LARC than the 15–24 age group. In 2014, the influence of age was minimal and was only exhibited for the women aged 25–34, who were 15% more likely to choose LARC than the younger 15-24 cohort.

Education showed some influence in 2003, with secondary educated women being 2.8 times more likely to use LARC as compared to those with no education. The variable had no influence in the other data sets. Residence also had very strong influence in one data set, 2014. Rural women were 20% more likely to use LARC than their urban counterparts.

Wealth exhibited different strengths ranging from low (p<.05), moderate (p<.01) to very strong (p<.001) in influence towards LARC. In 2003 the influence was moderate for women of lower wealth, who were 55% less likely to choose LARC than their wealthier counterparts. For women of medium wealth, the influence was low and they were 42% less likely to use LARC than the wealthier women. In 2008, wealth showed moderate influence in women of lower wealth, who were 55% less likely to choose LARC than women in the higher wealth category. In 2014, wealth showed very strong predictive ability for women of lower wealth, who were 26% less likely to use LARC than women with higher wealth status. Moderate influence was seen for women of medium wealth, who were 20% less likely to choose LARC when viewed against women of higher wealth.

Parity had low or very strong influence towards LARC use. In 2003, the influence was low, showing that women with 1-2 children were 3.6 times more likely to choose LARC than those with no children. In 2008, influence was again low, showing women with 1–2 children as three times more likely to choose LARC in relation to those with no children, while those with 3–4 children were 3.3 times more likely to use LARC than those without children. In 2014, the influence was very strong, showing those with 1–2 children as 11 times more likely to choose LARC than nulliparous women, while those with 3–4 children were 12 times more likely to choose LARC over those without children. Women with five or more children were 13 times more likely to choose LARC than those without children.

## Discussion

The study shows LARC having very low usage initially, before a big shift in use from other modern towards LARC between 2008/09 and 2014. The period of increased use coincided with LARC promotion campaigns that were conducted in Kenya and several African countries, which generated great demand by advancing LARC as the longer lasting, safer and more effective methods. The demand created was met by a steady supply of the methods. The implant has had higher uptake than the IUD, suggesting higher acceptability of the method, which may be due to ease and method of insertion, length of efficacy or preference for hormonal methods ^7^. The increase in LARC use was seen in many sub-Saharan African countries but the uptake of the implant in Kenya was the highest in the world at 18% prevalence as of 2017 ^19^.

The network of Tunza clinics in Kenya also had a project to increase LARC uptake through demand generation between 2009 and 2014. The project increased implant uptake by around 12% and IUD uptake by 5% within Tunza clinics ^20^. The Tupange project also scaled up use of implants in five urban centers over a five-year period ^21^. Overall, the relaunch of the Kenyan family planning program and launch of FP2020 in 2011, as well as the development of FP CIP 2012–2016 ^22^, aided the increase in LARC nationally.

The uptake of the IUD is low in Kenya in spite of the aggressive LARC campaigns, mostly because of rumors and misconception about their convenience and effectiveness as well as provider attitudes and bias ^23^. Of concern is its suitability for certain populations; for example, young women and women with a tendency towards multiple partners. The situation was different in the 1980s and it commanded a good share of the method mix in Kenya at 31% but thereafter started declining and is currently heavily underutilized ^23^.

Influence of age on LARC use was seen in all the data sets. LARC use was initially seen to be more common among women of at least 35 years, who are traditionally limiters as they have mostly attained their desired number of children and do not want more children. However, globally LARC was recommended for adolescents and young women ^24^ and those who were spacing, as fertility returned soon after the method was discontinued ^25^. Other studies have also associated age with LARC use ^26^, while others have found it insignificant ^27^.

As of 2014 in Kenya, a shift had occurred such that the leading users of LARC were now 25–34-year-olds, who are also the majority users of modern contraception, fulfilling the huge demand for spacing births. However, IUD use in Kenya shows dominance by the over 35s, who are limiting births. As expected, low LARC use was seen among the 15–24-year-olds and the reason for this may be their general lower use of modern contraception and their tendency towards short term methods because they are cautious about using methods that may interfere with their future fertility intentions ^28^. The group showed major increases in LARC use from 3% in 2008/09 to 20% in 2014 ^1, 29^.

This study showed LARC use was more prominent among women of higher socio-economic status, perhaps because of their better exposure and access to contraceptive information and services. Other studies have found LARC to increase with education ^26^ and wealth ^30, 31^, while others did not find any effects from education ^27^. LARC methods are generally more available in urban areas and a contributing factor is the lack of trained providers in rural and low-income areas to handle insertions and removal ^27^. For the IUD, the issue of insertion is sensitive and must be done correctly for a user to be satisfied and continue using it for the intended period. This contributes to high discontinuation rates for the IUD ^32^.

Analysis based on region showed that the Central region and Nairobi had the biggest shares of LARC against other modern methods. However, between 2008/09 and 2014, a shift was seen with regions with previously low contraception rates registering big surges in LARC uptake and some even overtaking the previous high contraception rate regions. The Western region was seen to overtake Central and Nairobi to have the biggest share of implant use among modern method usage. The North Eastern region, the region with the lowest contraceptive prevalence in Kenya, is also seen to be making good strides in terms of its proportion of LARC against other modern methods.

The reproductive goals of a woman are about the number and timing of the children she desires to have and the method she chooses is supposed to enhance the goal of either spacing or limiting births. This was emphasized by the strong influence seen from the number of living children, with LARC use increasing with the number of children. A possible explanation is that higher parity may expose a woman to a lot of information and experience about contraception during prenatal and postnatal clinics ^31, 33^.

Levels of LARC use seen in the study are low when viewed against its many contraceptive and non-contraceptive benefits. One barrier to LARC uptake is myths and fears of side effects. Qualitative studies reveal that among the believed side effects for the IUD is that it causes cancer, while other women think LARC will interfere with their future fertility, hence do not opt for them until they have attained their desired number of children ^27^. This study complements this view as analysis on fertility desire showed most LARC users are limiters.

### Policy and program implications for Kenya

The high demand shown for LARC should be accompanied by efforts to address barriers in the supply chain by reducing commodity stock outs.

LARC needs more funding than short term methods and with reduced donor funds, sustainable financing needs to be secured and ringfenced. However, LARC are cost-effective in the long term because resupplies and clinic visits needed are fewer.

LARC are provider dependent methods and the rising demand for them should be accompanied by investment in quality services for insertion and removal procedures. This might improve the acceptability and continuation of the IUD.

High demand for implants was evident among 15–24-year-old women. Given that this group is the fastest growing within the reproductive age, it is vital that easy access to LARC information and services is available for them.

The rise in the use of implants among women aged 15–24 has been accompanied by a huge decline in condom use, which may mean little or no protection against HIV/AIDS and other sexually transmitted infections. Perhaps use of condoms can be recommended alongside LARC for this group depending on the need.

The slow growth of IUD uptake suggests the challenges of insertion and side effects abound and calls for a change of strategy borrowed from regions that have higher IUD prevalence.

Large proportions of those aged 25–34 using LARC indicates the success of the policy shift to promoting LARC among spacers, while the increase in uptake among those aged 15–24 also shows acceptability among young, nulliparous women.

The huge uptake in the Western region and other regions that previously had low LARC use indicates the success of the LARC promotion campaigns and may point to some major changes in strategy in the promotion of LARC, which can guide family planning program managers in the regions where uptake is not as high.

Number of children has shown an increasingly strong influence on the choice of LARC and the share of spacers using LARC is increasing. Service providers need to package contraceptive counselling information to appeal to both spacers and limiters in future attempts to increase demand for LARC and especially for IUDs.

In general, results suggest contraceptive users are using methods that are suitable for fulfilling their fertility desires but the large disparities between proportions using LARC against those using other modern methods reveal a huge untapped potential for LARC uptake.

## Conclusions

The study has compared use of LARC to that of other modern contraception and documented how it has evolved from very low to significant shares in modern contraceptive use and the factors that have influenced its use over different time settings. It has established huge unexploited potential for more LARC uptake based on the identified predictors of its use. The data will be useful to inform strategies to improve LARC use and help Kenya increase mCPR and reduce unwanted fertility, unsafe abortions and unmet need for contraception.

Scaling up of LARC uptake is critical to deal with issues of poor user adherence, incorrect and inconsistent use and method failure that characterize short acting contraception, resulting in increased unintended pregnancies, incidences of unsafe abortions and maternal and infant mortality. Strategies are needed to provide accurate information to counteract rumors and misconceptions surrounding the IUD to increase its uptake.

## Data availability

### Source data

The Women Individual Recode datasets from the Kenya Demographic and Health Survey 2003, 2008/09 and 2014 were used for this study are available from the MEASURE DHS repository, ( http://www.measuredhs.com). Access to the dataset requires registration and is granted to those that wish to use the data for legitimate research purposes. A guide for how to apply for dataset access is available at: https://dhsprogram.com/data/Access-Instructions.cfm.

## References

[1] KNBS: Kenya Demographic and Health Survey 2014. Kenya National Bureau of Statistics and ICF MACRO.2015. Reference Source

[2] KNBS: Kenya Demographic and Health Survey 2003. Kenya National Bureau of Statistics and ICF MACRO.2004. Reference Source

[3] New JR Cahill N Stover J : Levels and trends in contraceptive prevalence, unmet need, and demand for family planning for 29 states and union territories in India: a modelling study using the Family Planning Estimation Tool. *Lancet Glob Health.* 2017;5(3):e350–e358. 10.1016/S2214-109X(17)30033-5 28193400

[4] Health Policy Project: Impact Now Model: Estimating the Health and Economic Impacts of Family Planning Use. Health Policy Project (HPP), United States Agency for International Development (USAID), Marie Stopes International (MSI) and Futures Group. Health Policy Project.2014. Reference Source

[5] KNBS: Kenya Population and Housing Census: Vol III. Distribution of Population by Age and Sex. Kenya National Bureau of Statistics.2019. Reference Source

[6] Shoupe D : LARC methods: entering a new age of contraception and reproductive health. *Contracept Reprod Med.* 2016;1:4. 10.1186/s40834-016-0011-8 29201394 PMC5675060

[7] Ngo TD Nuccio O Pereira SK : Evaluating a LARC Expansion Program in 14 Sub-Saharan African Countries: A Service Delivery Model for Meeting FP2020 Goals. *Matern Child Health J.* 2017;21(9):1734–1743. 10.1007/s10995-016-2014-0 27154524 PMC5569118

[8] Tsui AO Brown W Li Q : Contraceptive Practice in Sub-Saharan Africa. *Popul Dev Rev.* 2017;43(Suppl Suppl 1):166–191. 10.1111/padr.12051 29081552 PMC5658050

[9] Blumenthal PD Voedisch A Gemzell-Danielsson K : Strategies to prevent unintended pregnancy: increasing use of long-acting reversible contraception. *Hum Reprod Update.* 2011;17(1):121–37. 10.1093/humupd/dmq026 20634208

[10] Trussel J Hassan F Lowin J : Achieving cost-neutrality with long-acting reversible contraceptive methods. *Contraception.* 2015;91(1):49–56. 10.1016/j.contraception.2014.08.011 25282161 PMC4268022

[11] Bradley S Croft T Rutstein S : The impact of contraceptive failure on unintended births and induced abortions: Estimates and strategies for reduction. *DHS analytical studies 22.* ICF Macro, Calverton, Maryland.2011. Reference Source

[12] Higgins JA : Celebration meets caution: LARC's boons, potential busts, and the benefits of a reproductive justice approach. *Contraception.* 2014;89(4):237–241. 10.1016/j.contraception.2014.01.027 24582293 PMC4251590

[13] Ministry of Health: National Family Planning Costed Implementation Plan 2017-2020. Ministry of Health, Kenya.2017. Reference Source

[14] Hubacher D Spector H Monteith C : Long-acting reversible contraceptive acceptability and unintended pregnancy among women presenting for short-acting methods: a randomized patient preference trial. *Am J Obstet Gynecol.* 2017;216(2):101–109. 10.1016/j.ajog.2016.08.033 27662799 PMC5479328

[15] Secura GM Allsworth JE Madden T : The Contraceptive CHOICE Project: reducing barriers to long-acting reversible contraception. *Am J Obstet Gynecol.* 2010;203(2):115.e1–7. 10.1016/j.ajog.2010.04.017 20541171 PMC2910826

[16] Central Bureau of Statistics - CBS/Kenya, Ministry of Health - MOH/Kenya, and ORC Macro: Kenya Demographic and Health Survey 2003. Calverton, Maryland, USA: CBS MOH, and ORC Macro.2004. Reference Source

[17] Kenya National Bureau of Statistics - KNBS, National AIDS Control Council/Kenya, National AIDS/STD Control Programme/Kenya, Ministry of Public Health and Sanitation/Kenya, and Kenya Medical Research Institute: Kenya Demographic and Health Survey 2008-09. Calverton, Maryland, USA: KNBS and ICF Macro.2010. Reference Source

[18] Kenya National Bureau of Statistics, Ministry of Health/Kenya, National AIDS Control Council/Kenya, Kenya Medical Research Institute, and National Council for Population and Development/Kenya: Kenya Demographic and Health Survey 2014. Rockville, MD USA.2015. Reference Source

[19] Jacobstein R : Liftoff: The Blossoming of Contraceptive Implant Use in Africa. *Glob Health Sci Pract.* 2018;6(1):17–39. 10.9745/GHSP-D-17-00396 29559495 PMC5878070

[20] PSI: Increasing LARC uptake in Kenya through Improved Demand Creation Strategies and Provider Support. Best Practices from the Women’s Health Project 2009-2014. Population Services International.2015. Reference Source

[21] Keyonzo N Nyachae P Kagwe P : From Project to Program: Tupange's Experience with Scaling Up Family Planning Interventions in Urban Kenya. *Reprod Health Matters.* 2015;23(45):103–13. 10.1016/j.rhm.2015.06.010 26278838

[22] Ministry of Health: National Family Planning Costed Implementation Plan 2012-2016. Ministry of Health, Kenya.2012. Reference Source

[23] The ACQUIRE Project/EngenderHealth: Revitalizing the IUD in Kenya. Acquiring Knowledge.2006;2.

[24] Chandra-Mouli V Mccarraher DR Phillips SJ : Contraception for adolescents in low and middle income countries: needs, barriers, and access. *Reprod Health.* 2014;11(1):1. 10.1186/1742-4755-11-1 24383405 PMC3882494

[25] Joshi R Khadilkar S Patel M : Global trends in use of long-acting reversible and permanent methods of contraception: Seeking a balance. *Int J Gynaecol Obstet.* 2015;131(S1):S60–3. 10.1016/j.ijgo.2015.04.024 26433510

[26] Biza N Abdu M Reddy PPS : Long acting reversible contraceptive use and associated factors among contraceptive users in Amhara Region, Ethiopia, 2016. A community Based cross sectional study. *Med Res Chron.* 2017;4(5):469–480.

[27] Tibaijuka L Odongo R Welikhe E : Factors influencing use of long-acting versus short-acting contraceptive methods among reproductive-age women in a resource-limited setting. *BMC Womens Health.* 2017;17:25. 10.1186/s12905-017-0382-2 28376779 PMC5379613

[28] Payne JB Sundstrom B DeMaria AL : A Qualitative Study of Young Women's Beliefs About Intrauterine Devices: Fear of Infertility. *J Midwifery Womens Health.* 2016;61(4):482–8. 10.1111/jmwh.12425 26971722

[29] KNBS: Kenya Demographic and Health Survey 2008/09. Kenya National Bureau of Statistics and ICF MACRO.2010. Reference Source

[30] Adedini SA Omisakin OA Somefun OD : Trends, patterns and determinants of long-acting reversible methods of contraception among women in sub-Saharan Africa. *PLoS One.* 2019;14(6): e0217574. 10.1371/journal.pone.0217574 31163050 PMC6548375

[31] Bhandari R Pokhrel KN Gabrielle N : Long acting reversible contraception use and associated factors among married women of reproductive age in Nepal. *PLoS One.* 2019;14(3): e0214590. 10.1371/journal.pone.0214590 30921403 PMC6438478

[32] Wildemeersch D : Intrauterine contraceptives that do not fit well contribute to early discontinuation. *Eur J Contracept Reprod Health Care.* 2011;16(2):135–141. 10.3109/13625187.2010.546533 21281098

[33] Anguzu R Sempeera H Sekandi JN : High parity predicts use of long-acting reversible contraceptives in the extended postpartum period among women in rural Uganda. *Contracept Reprod Med.* 2018;3:6. 10.1186/s40834-018-0059-8 29760943 PMC5941484

