# Supplementary material for: Trends and factors associated with long-acting reversible contraception in Kenya
Source: F1000Res. 2020 May 20;9:382. [Version 1] doi: 10.12688/f1000research.23857.1 (PMC9152462; doi:10.12688/f1000research.23857.1)
Supplement: Peer review history

# Peer review: Reviewer response for version 1

**DOI:** 10.5256/f1000research.26324.r138102

**Published:** 2022-05-30

**Authors:** John Cleland

**Affiliations:** 1 Faculty of Epidemiology and Population Health, London School of Hygieneand Tropical Medicine, London, UK

The increasing uptake of LARCs in Kenya, and in many other African countries, is an important development and a paper on factors that are associated with use of these methods is welcome. The sharp focus on method choice (LARCs versus other modern methods) is appropriate. The overall message of the paper is clear but several problems need to be addressed.

1. Your handling of region should be reconsidered. In Tables 1 and 2, you show results for high versus low use regions but differences are minor and not statistically significant. However in figures 6, 7 and 8 you show results for Kenya’s 8 regions. I cannot understand why results for these 8 regions are not included in tables 1 and 2. My suggestion is that you do include them and omit the low/high use characterisation of region.
2. A total of nine figures seems to me excessive. All the results in these 9 figures could be compressed into a single table. It would look like table 1 but instead of % LARC and % other modern, it would show % implant and % IUD. This would have the advantage of showing results across the three surveys for variables such as wealth and urban-rural residence which are excluded from the figures. Please consider this option.
3. Even if you insist on retention of figures, some of them should be re-designed. Figures 2-4 and 9 shows profiles of users. It would be more informative to re-design as in figure 1. For instance in figure 4, it is impossible to tell whether the very small % of uneducated women using implants is due to an aversion to implants among these women or to the fact that there are very few uneducated modern method users. A line graph as in figure 1 with denominators of all modern method users of specified educational status would be better. Whether you follow the suggestion in (2) or re-design figures, the text would need to be amended.

Minor points

1. Headings of tables and figures should be more precise. For instance the title for table 1 should be “Percentage of all modern method users who use long-acting and other methods by background characteristics, 2003-2014.
2. In table 2 I see no need to show B values and standard errors. It is also more customary to label EXP B as Odds ratios. In the footnote, the reference category is Other methods NOT LARCs.
3. The Discussion would benefit from consideration of the relevant Kenyan evidence about method-specific beliefs and method choice e.g., Odwe *et al*., (2021) ^1^; Obare *et al.,* (2020) ^2^; Machiyama *et al.,* (2018) ^3^.
4. Some brief discussion concerning the introduction to Kenya of the levonorgestrel intrauterine system should be added.

Is the work clearly and accurately presented and does it cite the current literature?

Partly

If applicable, is the statistical analysis and its interpretation appropriate?

Partly

Are all the source data underlying the results available to ensure full reproducibility?

Yes

Is the study design appropriate and is the work technically sound?

Partly

Are the conclusions drawn adequately supported by the results?

Yes

Are sufficient details of methods and analysis provided to allow replication by others?

Yes

Reviewer Expertise:

Social demography with focus on fertility and contraception

I confirm that I have read this submission and believe that I have an appropriate level of expertise to confirm that it is of an acceptable scientific standard, however I have significant reservations, as outlined above.

## References

[1] : Method-specific beliefs and subsequent contraceptive method choice: Results from a longitudinal study in urban and rural Kenya. *PLoS One* .2021;16(6) : 10.1371/journal.pone.0252977 e0252977 10.1371/journal.pone.0252977 34143804PMC8213138

[2] : Exploring the Demand-Side Factors Associated with the Use of Implants in Kenya. *Stud Fam Plann* .51(2) : 10.1111/sifp.12117 119-137 10.1111/sifp.12117 32515508

[3] : Women's attitudes and beliefs towards specific contraceptive methods in Bangladesh and Kenya. *Reprod Health* .2018;15(1) : 10.1186/s12978-018-0514-7 75 10.1186/s12978-018-0514-7 29739429PMC5941610

# Peer review: Reviewer response for version 1

**DOI:** 10.5256/f1000research.26324.r138105

**Published:** 2022-05-18

**Authors:** Kate Cheney

**Affiliations:** 1 Faculty of Medicine and Health, University of Sydney, New South Wales, Australia

Thank you for submitting your manuscript titled: Trends and factors associated with long-acting reversible contraception in Kenya

 This is interesting and important work for the reader Understanding the barriers to the LARC options are essential (not just in developed countries) to move forward to proving LARC methods to those who wish it.

**Abstract:**

 No comments.

**Introduction:**

- Is this a government or health agenda?
- Can you provide a reference of LARC?
- Not sure you need the word HUGH before female population?
- Can you reference a quick example of heath and social benefits for the reader?
- Is there data about postpartum contraception opportunities? This is often a time of engagement with health midwives and doctors.
- The paragraph starting with "A challenge in the access..." is a bit unclear for the reader - can this be clarified a little please?
- There are other partners outside family planning programs, like postpartum, so would be good to include all opportunities. 
- Objective: clear

**Methods:**

- Methods - what is ICF?

**Results:**

- There are a great many tables but are they all needed?
- Parity is very interesting any reason for that? Is contraception well provided in the postpartum period, or is it free then?

**Discussion:**

- What are Tunza clinics? Could you explain a little, no all readers will know about Kenya or this.
- Could the statement about omen with tendency to multiple partner be better expressed?
- Does type of IUD matter? i.e. copper, mirena, etc.
- What is it about those regions where LARC use is more common?
- Where is the ref about reproductive goals of women, some may have no goals or plans, or feel they have no choice?
- Who inserts LARC, are all workforce being included? i.e. nurses and midwives too.
- Are health professionals trained in LARC?
- Does it come down to cost, local support and supplies?

**Conclusion:** 

 Thank you.

 I am not able to assess the statistical  analysis

Is the work clearly and accurately presented and does it cite the current literature?

Yes

If applicable, is the statistical analysis and its interpretation appropriate?

I cannot comment. A qualified statistician is required.

Are all the source data underlying the results available to ensure full reproducibility?

Yes

Is the study design appropriate and is the work technically sound?

Yes

Are the conclusions drawn adequately supported by the results?

Yes

Are sufficient details of methods and analysis provided to allow replication by others?

Yes

Reviewer Expertise:

Pregnancy planning, contraception, preconception, postpartum contraception, nursing, midwifery

I confirm that I have read this submission and believe that I have an appropriate level of expertise to confirm that it is of an acceptable scientific standard, however I have significant reservations, as outlined above.

# Response

**Published:** 2022-05-18

**Authors:** Margaret Kungu

**Affiliations:** University of Nairobi, Kenya

Thank you Kate for the very insightful comments which will help improve the paper further.

 Below are immediate responses as I work on an improved revision.

- Is this a government or health agenda? **It is both as the Government has been encouraging uptake of FP to rpomote women health and attain UHC**
- Can you provide a reference of LARC? **Yes, I will.**
- Not sure you need the word HUGH before female population? **Huge - It was an emphasis on the size.**
- Can you reference a quick example of heath and social benefits for the reader? **Yes**
- Is there data about postpartum contraception opportunities? This is often a time of engagement with health midwives and doctors. **The data is not available in KDHS in this context.**
- The paragraph starting with "A challenge in the access..." is a bit unclear for the reader - can this be clarified a little please? **It is about expanding the method mix so that injection is not the dominant option. I will revise in the final version.**

There are other partners outside family planning programs, like postpartum, so would be good to include all opportunities. **Yes in line with the focus of the study (trends and factors associated with use of LARC) and available data.**

- Methods - what is ICF? **ICF Macro are the administrators of the DHS worldwide program.**
- There are a great many tables but are they all needed? **Yes . The tables are only 2 not many but the graphs are quite a number to show LARC use by different key socio-economic variables hence they are necessary.**
- Parity is very interesting any reason for that? Is contraception well provided in the postpartum period, or is it free then? **It may be because LARCS were previously encouraged among those limiting children but not spacers. Contraception is freely available in Kenya especially for postpartum clients**

**Discussion:**

- What are Tunza clinics? Could you explain a little, no all readers will know about Kenya or this. **Tunza clinics were an Non- Governmental initiative for scaling up family planning. I will clarify in the final version.**
- Could the statement about women with tendency to multiple partner be better expressed? **Yes in the final version**.
- Does type of IUD matter? i.e. copper, mirena, etc. **Yes but KDHS does not have the data on types.**
- What is it about those regions where LARC use is more common? **This is where the FP campaigns to market LARC were very successful.**
- Where is the ref about reproductive goals of women, some may have no goals or plans, or feel they have no choice? **It will be incorporated if available.**
- Who inserts LARC, are all workforce being included? i.e. nurses and midwives too. **LARCs are inserted by nurses.**
- Are health professionals trained in LARC? **Yes very well.**
- Does it come down to cost, local support and supplies? **Cost is not a major issue but maybe acceptance and supplies in some far flung areas. **

# Peer review: Reviewer response for version 1

**DOI:** 10.5256/f1000research.26324.r63727

**Published:** 2020-06-15

**Authors:** Jeffrey F. Peipert

**Affiliations:** 1 Department of Obstetrics and Gynecology, Indiana University School of Medicine, Indianapolis, IN, USA

I enjoyed reading the article, *Trends and factors associated with long-acting reversible contraception in Kenya,* by Dr. Kungu and colleagues. The article is well written and provides useful information for researchers and clinicians interested in providing comprehensive contraception care globally. 

 The study is a secondary analysis of 3 waves of the Kenya Demographic Health Survey. I am not an expert in large survey design and analysis; thus, my review will focus on the usefulness of the information provided. It was encouraging to read that LARC use is “picking up rapidly” in Kenya. The authors group LARC together (IUDs and implants) and separate out implants and IUDs in the text, but they do not cover IUD types (copper and hormonal). This would be useful information to the reader. I believe hormonal IUDs are not as available as copper in Kenya. Is this true? Has it changed over time? There are some sections of the manuscript that lack balance (advantages/disadvantages of LARC). For example, 4 ^th^ paragraph of the INTRO: the authors highlight the many advantages of LARC, but make no mention of the disadvantages (e.g. irregular bleeding with the implant and heavier bleeding with the copper IUD).

 The Discussion provided some interesting information. I had no idea that the uptake of the implant in Kenya was “the highest in the world.” In the third paragraph of the Discussion, the authors mention the “rumors and misconceptions.” What are the most common misconceptions? Do women believe the IUD is linked to infertility? Looks like this is mentioned in paragraph 5; is this misconception limited to younger women? There is some discussion of these myths in paragraph 9 (e.g. mention of cancer).

 In the discussion of age and LARC, it may be helpful to stratify. In many populations, the implant is more popular in young women, and the IUD is more accepted in older women. 

 The authors also comment on the decline in condom use. Has there been an epidemic of STIs as a result? Is there any evidence of a detrimental effect on the population? I do agree with a renewed emphasis on dual-use (barrier plus effective method) for STI and pregnancy prevention.

 Overall, I found the Discussion a bit long, wordy, and somewhat disorganized. Avoid one-sentence paragraphs. Group topics (age) together. It would benefit from consolidation and being more concise.

   

**Specific comments:**

  

- ABSTRACT: Background: I do not think you can say there is NO risk of incorrect use. Unrecognized expulsion of an IUD is an example. Methods: You might provide the percentage of the population that are “currently using modern contraceptive methods” here. Results: It would be helpful to provide some data, effect estimates, and some confidence intervals. Which factors have the biggest effect estimate on the association with LARC use?
- INTRO: 4 ^th^ paragraph, last sentence: I did not understand “they arrive at costs of _____.” Is this cost savings? I assume the cost of the methods are estimates of the true cost. Please explain more clearly.
- METHODS: Please state the type of regression used (logistic regression) in the text How did you assess confounding? Did you look for interactions?
- RESULTS: I would prefer an adjusted analysis that shows the OR and 95% CI. I do not think we need the B, SE in Table 2. A simplified table showing the effect estimates and confidence intervals would be better.
- DISCUSSION: Comments above.
- There’s LOTS of information, tables, and Figures. I trust the editor will decide if all of these are needed.

 

 Overall, a well done report.

Is the work clearly and accurately presented and does it cite the current literature?

Yes

If applicable, is the statistical analysis and its interpretation appropriate?

Partly

Are all the source data underlying the results available to ensure full reproducibility?

Yes

Is the study design appropriate and is the work technically sound?

Yes

Are the conclusions drawn adequately supported by the results?

Yes

Are sufficient details of methods and analysis provided to allow replication by others?

Yes

Reviewer Expertise:

Women's Health; family planning; contraception; sexually transmitted infections; public health

I confirm that I have read this submission and believe that I have an appropriate level of expertise to confirm that it is of an acceptable scientific standard.

# Response

**Published:** 2020-06-15

**Authors:** Margaret Kungu

**Affiliations:** University of Nairobi, Kenya

As the corresponding author, I appreciate the expert, thorough, and very useful comments of the reviewer and will address them in the revised article so as to improve it for readers.
